# Health Information Seeking on the Internet Among Patients With and Without Cancer in a Region Affected by the 2011 Fukushima Triple Disaster: Cross-Sectional Study

**DOI:** 10.2196/49897

**Published:** 2024-08-21

**Authors:** Yudai Kaneda, Akihiko Ozaki, Michio Murakami, Toyoaki Sawano, Shuhei Nomura, Divya Bhandari, Hiroaki Saito, Masaharu Tsubokura, Kazue Yamaoka, Yoshinori Nakata, Manabu Tsukada, Hiromichi Ohira

**Affiliations:** 1 School of Medicine Hokkaido University Sapporo Japan; 2 Research Center for Community Health Minamisoma Municipal General Hospital Minamisoma Japan; 3 Breast and Thyroid Center Jyoban Hospital of Tokiwa Foundation Iwaki Japan; 4 Department of Thyroid and Endocrinology Fukushima Medical University Fukushima Japan; 5 Department of Health Risk Communication Fukushima Medical University Fukushima Japan; 6 Center for Infectious Disease Education and Research Osaka University Suita Japan; 7 Department of Surgery Jyoban Hospital of Tokiwa Foundation Iwaki Japan; 8 Department of Radiation Health Management Fukushima Medical University School of Medicine Fukushima Japan; 9 Keio University Global Research Institute (KGRI) Tokyo Japan; 10 Department of Internal Medicine Soma Central Hospital Soma Japan; 11 Graduate School of Public Health Teikyo University Tokyo Japan; 12 Tetsuyu Clinical Research Center Tetsuyu Healthcare Holdings Pte Ltd. Tokyo Japan; 13 Department of Surgery Minamisoma Municipal General Hospital Minamisoma Japan

**Keywords:** health information, patients with cancer, disaster, Japan, internet, patient with cancer, cancer, internet health, mobile phone

## Abstract

**Background:**

Health information seeking via the internet among patients with cancer in disaster-affected areas is underresearched.

**Objective:**

This study aims not only to assess the extent and means of web-based health information seeking among patients with cancer living in the disaster-affected area of the 2011 Fukushima triple disaster but also to compare these patterns with those without cancer, identifying distinct and shared factors influencing their web-based health information behaviors.

**Methods:**

We surveyed 404 patients (263 with and 141 without cancer) from the surgery department outpatient office at Minamisoma Municipal General Hospital, from October 2016 to January 2017. The survey included self-administered questions on internet and digital device use. Descriptive analyses were performed to examine the use patterns of digital devices and the internet and their impact on health information seeking across different age groups of patients with and without cancer. Multivariable logistic regression was used to examine factors associated with web-based health information seeking, stratifying by cancer diagnosis.

**Results:**

The proportion of participants who sought health information on the internet was comparable between patients with cancer and patients without cancer (19% vs 17.4%; *P*=.71). Digital device use varied significantly with age, with peak smartphone use occurring among the youngest cohorts for both groups. Multivariable logistic regression revealed that patients with cancer using smartphones or tablets daily were significantly more likely to gather web-based health information (odds ratio [OR] for smartphones 3.73, 95% CI 1.58-8.80; OR for tablets 5.08, 95% CI 1.27-20.35). Trust in institutional websites also significantly influenced web-based health information gathering among patients with cancer (OR 2.87, 95% CI 1.13-7.25). Conversely, among patients without cancer, unemployment was associated with a lower likelihood of seeking web-based health information (OR 0.26, 95% CI 0.08-0.85), whereas trust in both institutional and personal websites significantly increased this likelihood (OR for institutional websites 6.76, 95% CI 2.19-20.88; OR for personal websites 6.97, 95% CI 1.49-32.58).

**Conclusions:**

This study reveals that a small proportion of both patients with cancer and patients without cancer engage in health information seeking via the internet, influenced by age, digital device use, and trust in institutional websites. Given the growing prevalence of digital literacy, strategies to enhance accessible and reliable web-based health information should be developed, particularly for patients with cancer in postdisaster settings. Future efforts should focus on tailored health communication strategies that address the unique needs of these populations.

## Introduction

Individuals diagnosed with cancer require a wealth of health information to effectively manage their daily lives and the course of their treatment [1]. Seeking such information has been shown to yield a magnitude of beneficial health effects within this patient group [2,3]. Nonmedical information sources, especially those available via the internet, have become increasingly significant to these individuals in recent years [4]. In the United States, there has been a notable rise in patients with cancer seeking web-based health information, with the percentage increasing from 10% in 2005 to 62% in 2018 [5,6]. However, the frequency of internet use among this patient group varies widely based on several sociodemographic factors including age, gender, educational level, and socioeconomic status [4,7-9].

Not only do individual characteristics impact the extent of health information seeking via the internet but external circumstances also play a vital role [10]. Crises and disasters serve as a particularly relevant context to explore this phenomenon due to the expansive range and magnitude of health information needs they instigate among patients with cancer, as evidenced during events such as the 2011 Japan triple disaster and the recent COVID-19 pandemic [11-15]. Furthermore, there is emerging evidence indicating the internet’s efficacy and growing popularity as a medium for transmitting health information to patients with cancer [16,17]. In summary, the internet is a highly convenient and useful tool to access health information among patients with cancer. Medical institution staff often face overwhelming responses from the sick and injured, making the internet a valuable resource. However, the accuracy and reliability of web-based health information have been subject to debate, especially in the acute phase [10,16,18]. It is worth noting that there is a lack of empirical studies investigating health information seeking specifically through the internet for patients with cancer, especially in non–COVID-19 contexts [19].

**Figure 1:**
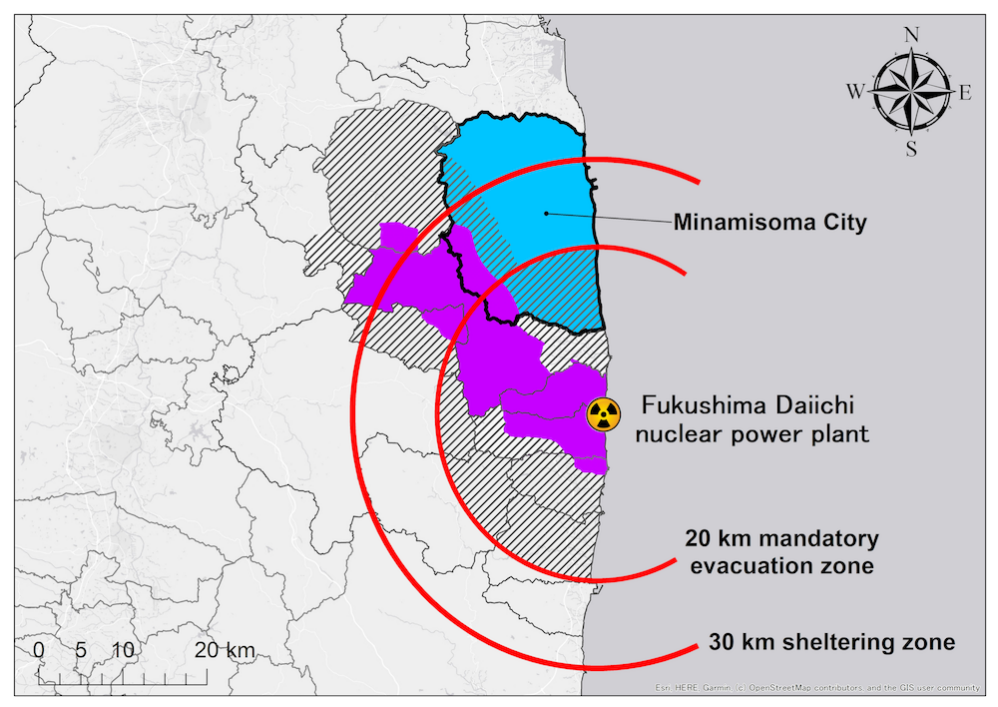
The geographical location of Minamisoma City and the FDNPP, with the transition of evacuation zones over time. Minamisoma City is located 14-38 km to the north of the FDNPP. Following the earthquake and subsequent tsunami on March 11, 2011, an initial hydrogen explosion occurred at reactor 1 of the nuclear power plant on March 12, 2011, and the areas within 20 and 30 km radius of the power plant were designated as mandatory evacuation and sheltering zones, respectively. The evacuation zone was expanded (in border, red) on April 22, 2011, and again at later dates, lifting orders from certain places (in border) with the progress of restoration work; however, the order remained in effect primarily in areas with severe contamination near the power plant (in red), as of June 1, 2019. Minamisoma Municipal General Hospital (triangle) is located 23 km north of the power plant, which is within the sheltering zone. Permission for the use of this image has been granted by ESRI Japan Corporation. This figure was reproduced from our previous open-access study under the CC BY-NC license [25]. FDNPP: Fukushima Daiichi nuclear power plant.

In this sense, the 2011 Japan triple disaster provides an unprecedented case study for examining the use of internet-based health information in disaster settings. On March 11, 2011, a significant earthquake triggered a tsunami, which in turn precipitated a nuclear accident at the Fukushima Daiichi nuclear power plant (FDNPP; Figure 1 [20]). The ensuing fallout significantly impacted the nearby city of Minamisoma, situated between 14 and 38 km north of the FDNPP [21]. In the aftermath of the accident, sections of Minamisoma City were designed as mandatory evacuation and sheltering zones by the central government. This order, the first issued on March 12, 2011, and later revised [22], precipitated a rapid depopulation and an acceleration in the aging of the city’s population [21,23]. Irrespective of radiation exposure as a secondary consequence of the disaster, including evacuations, an increase in various secondary health issues, such as the increased prevalence of chronic diseases including diabetes and delays in hospital visits, has been observed among the residents [24].

Simultaneously, the disaster had a noticeable impact on the societal structures of Minamisoma, weakening the community and familial support systems. This change was particularly pronounced in the context of health care, where the residents found themselves increasingly isolated and underserved [14]. Notably, the triple disaster also led to a reduction in the number of medical facilities within the city [25]. In light of these circumstances, the internet has emerged as a vital lifeline for residents, particularly for those diagnosed with cancer. The accessibility and convenience of web-based health information platforms have potentially increased their relevance in this health-challenged community.

Our initial investigation not only evaluated the scope of health information exposure (HIE) among the local patients with cancer at Minamisoma Municipal General Hospital (MMGH) but also included a comparative analysis with patients without cancer to assess differences in HIE between the two groups in the aftermath of the disaster. We found that most participants were regularly exposed to health information [20]. However, our study did not specifically explore HIE facilitated through the internet. We posit that using the residual data from our previous study could provide insight into additional queries in this follow-up study. First, foundational data on the internet use for health information seeking, an active and major form of HIE, such as frequency and the devices used to gain internet access, is vital for understanding the role of the internet in health information seeking. Second, identifying factors associated with health information seeking through the internet is a crucial investigative area for elucidating the demographics accessible and those inaccessible via the internet in postdisaster settings. Our particular interest is the impact of age on digital device use and internet access in these settings, given existing reports indicating a decrease in digital device use among older individuals in nondisaster settings [26].

The primary objectives of this study were to assess the extent and distinctive characteristics of health information seeking through the internet among the patients with cancer at MMGH, located in the area affected by the 2011 Fukushima triple disaster with a particular focus on the presence or absence of cohabitants. This is because it is plausible that family members living with the patient may have learned how to use the internet and have developed the habit of gathering health information on the internet.　We also examined the factors associated with health information seeking through the internet. We believe that the findings will aid health care professionals, media, and government entities in tailoring health information delivery to patients with cancer in the aftermath of disasters.

## Methods

### Settings and Participants

The study setting was a surgery department outpatient office at MMGH (Figure 1 [20]), which is located 23 km to the north of the FDNPP. MMGH is the central hospital of this and treats most of the patients with cancer in these areas. Details of the disaster experience, the subsequent recovery process, and the care of patients at MMGH and its surgical department can be obtained in our previously published papers [20,27].

Following a previous study [28], this study included all 404 adult patients (18 years old or older) among the total of 493 patients who visited this surgical department outpatient office and agreed to participate in the study from October 17, 2016, to January 31, 2017, with more details on the survey period and the characteristics of patients with cancer and patients without cancer are also available in our previously published paper [20]. The cohort consisted of 263 patients with cancer (median age 67 years; male 30%, n=79) and 141 patients without cancer (median age 63 years; male 46.1%, n=65) who agreed to participate in the study, providing a basis for a comparative analysis of health information seeking via the internet [20]. Individuals without consent or younger than 18 years of age were excluded from this study. As previously reported, to represent the population of 2 million people in Fukushima Prefecture with a 5% margin of error and a 95% CI, a sample size of 384 individuals is required [29]. The number of patients who participated in this study exceeded that amount. Given that the overall study project primarily sought to provide a comprehensive overview of HIE among both patients with and those without cancer in areas affected by disaster, we did not establish inclusion or exclusion criteria predicated on clinical variables among the patient with cancer cohort, encompassing disease stage or the nature of active treatments [20]. The logistic regression analysis was stratified by the presence or absence of cancer.

### Survey

We created the survey items by revising previous literature in accordance with the local and postdisaster context of cancer care in Minamisoma City [28,30,31]. Patients who visited the MMGH surgical outpatient department during the study period were included in the study. The survey comprised 3 sections: the first section covered items for health information gathering, internet use, and digital device use; the second section covered items for health status and attitudes toward health care; and the third section covered items for sociodemographic and disaster-related characteristics. In this study, we primarily focused on the answers to items for internet use, digital device use, and sociodemographic and disaster-related characteristics. The detailed survey complication process and outpatient operations were both described in our previous study [20].

### Variables

#### Primary Diagnosis and Sociodemographic Factors

When collecting the survey, participants’ primary disease was extracted from their medical records and classified as cancer (eg, breast cancer or gastric cancer) or noncancer (eg, trauma and inguinal hernia). Those who were assigned as patients with cancer had already been diagnosed at the time of recruitment into this study*.*

#### Use of the Internet and Digital Devices

Frequency of internet use was assessed with the item “How often do you use the internet?” (everyday, once every few days, once per week, once per month, or never). Further, among those who used the internet at least once per month, we evaluated the purposes by which they use the internet, with the item “For what purposes do you use the internet?” The patients responded whether they used the internet for general information seeking, health information seeking, social networking services, emailing, internet banking, and other purposes, using the 2-point scale (yes or no). Similarly, we assessed the daily use of digital devices with the item “Do you use the following digital devices every day?” for cellular phones (other than smartphones), smartphones, tablet devices, and personal computers. The variables are listed in the results table.

#### Health Information Seeking Using the Internet

We used the items “How often do you use the internet?” (everyday, once every few days, once per week, once per month, or never) and “For what purposes do you use the internet?” to assess the health information seeking using the internet. We combined the responses to these items and created a variable addressing the participants who use the internet at least once per month for health information seeking, “Health information seeking using the internet,” with a 2-point scale (yes or no).

#### Cohabitant Status

With regard to cohabitant status, we considered a partner, children, family members other than a partner and children, and any type of family member. Those who answered that they lived with these people before or after the earthquake were classified as “Living together with at least one family member either pre- or postdisaster.”

### Data Analysis

We performed 3 analyses. First, we made descriptive analyses for variables relating to the internet and digital devices both among patients with cancer and patients without cancer, namely the proportion of those who relied on each source of information, frequency of internet use, and the purposes of the internet use among the patients with cancer who seek information on the internet. Further, we evaluated a proportion of the patients who sought health information using the internet. We used the Mann-Whitney *U* test for continuous variables and the Fisher exact test or chi-square test for categorical variables, as appropriate, comparing patients with cancer and patients without cancer.

Second, to clarify whether there were any age effects on daily digital device use and health information–seeking behavior on the internet among patients with cancer, we calculated the proportion of those who were engaged in these behaviors, both patients with cancer and patients without cancer. The patients were then categorized according to their age with a 10-year age unit (49 years or younger, 50-59 years, 60-69 years, 70-79 years, and 80 years or older).

Third, to clarify the characteristics of patients with cancer who seek health information using the internet, we constructed a logistic regression model for the binary variable “Health information seeking using the internet.” As covariates, we considered all sociodemographic and health-related factors, and daily use of digital devices, using the backward stepwise variable selection method (inclusion criteria *P*=.05). The primary interest of this analysis was to detect any association between the exposure to health information and cohabitant status, and covariates other than cohabitant status were also examined in an exploratory manner. The covariates with a small number of participants were regrouped as necessary. We constructed the same model for patients without cancer for comparison. We estimated the variation inflation factor for the variables used in the final model to assess multi-collinearity, using the Stata command “Collin” [32].

As we aimed to examine whether a diagnosis of cancer influences health information seeking via the internet, “Health information seeking using the internet” was set as the primary outcome, with the remaining factors being the independent variables. All analyses were performed using Stata/IC 15.0 (StataCorp LLC).

### Ethical Considerations

This study received ethical approval from the MMGH ethics committee (approval 30-10) and Fukushima Medical University (approval 3064). Hospital staff trained for the study briefly explained it to patients who consented to participate and distributed the materials. They were notified that their data would be anonymized and used for academic purposes, and they were also informed that they could withdraw their consent at any time before the paper was submitted to journals. As such, participants in the original study consented to the use of their data obtained in the survey; thus, for this secondary analysis, which used the same data set as the original study, the original informed consent and Institutional Review Board approval sufficiently covered the use of anonymized data without requiring additional consent. No financial compensation was provided to participants, and the study adhered to stringent privacy and confidentiality measures to protect participant information.

## Results

Table 1 shows the participants’ reliance and use of the internet and digital devices. There was a significant difference in the frequency of internet use between patients with cancer and patients without cancer (*P*=.05); 39.9% (n=99) of the patients with cancer and 52.2% (n=72) of the patients without cancer used the internet at least once per month, respectively. In patients with cancer, the most popular purpose of internet use was seeking general information (n=85, 86%), followed by seeking health information (n=47, 47%) and emailing (n=33, 33%). In contrast, in patients without cancer, the most popular purpose was seeking general information (n=61, 85%), followed by the use of social networking services (n=30, 42%) and seeking health information (n=24, 33%). With regard to use of the digital devices, the proportion of patients who use smartphones on a daily basis was significantly smaller in patients with cancer than in patients without cancer (n=58, 22.1% vs n=61, 43.3%; *P*<.001). A proportion of the participants who sought health information on the internet did not differ between patients with cancer and patients without cancer (n=47, 19% vs n=24, 17.4%; *P*=.71).

Table 2 shows the proportions of the patients with daily digital device use and regular health information seeking on the internet in patients with cancer and those without cancer, with classifications of the patients by 10-year age unit. Among patients with cancer, daily cell phone use peaked at ages 70-79 years (n=39, 53%), while patients without cancer had the highest rates of use at ages 60-69 years (n=25, 66%). In addition, the percentage of tablet use peaked among patients with cancer aged 50-59 years (n=4, 9%), whereas the patients without cancer had the highest percentage of use among those aged 49 years or younger (n=6, 14%). The percentage of patients with cancer who gather health information on the internet peaked at age 49 years or younger (n=13, 46%), while among patients without cancer, the percentage of those who gather health information on the internet peaked at age 50-59 years (n=7, 33%). Personal computer use was highest among patients with cancer at age 49 years or younger (n=13, 46%), whereas it was highest among patients without cancer at age 50-59 years (n=8, 38%). The age groups with the highest percentage of each of the above devices were different for patients with cancer and patients without cancer, but for smartphone use, the highest percentage of both was 49 years old or younger (patients with cancer: n=23, 82%; patients without cancer: n=36, 86%).

**Table 1:** Participants’ reliance and use on the internet and digital devices.

			Patients without cancer (n=141)		Patients with cancer (n=263)	*P* value	
**Reliance on newsletter, n (%)**							.07
	Yes	0 (0)		6 (2.3)			
	No	141 (100)		257 (97.7)			
**Reliance on personal websites, n (%)**							.06
	Yes	10 (7.1)		8 (3)			
	No	131 (92.9)		255 (97)			
**Reliance on institutional websites, n (%)**							.23
	Yes	25 (17.7)		35 (13.3)			
	No	116 (82.3)		228 (86.7)			
**Reliance on social networking services, n (%)**							.37
	Yes	4 (2.8)		4 (1.5)			
	No	137 (97.2)		259 (98.5)			
**Frequency of the internet use^a^, n (%)**							.05
	Everyday	49 (35.5)		53 (21.4)			
	Once per 2 or 3 days	12 (8.7)		24 (9.7)			
	Once per week	7 (5.1)		12 (4.8)			
	Once per month	4 (2.9)		10 (4)			
	None	66 (47.8)		149 (60.1)			
**Purposes of internet use (among those who regularly use the internet)^b^, n (%)**							
	Seeking of general information	61 (84.7)		85 (85.9)		.84	
	Seeking of health information	24 (33.3)		47 (47.5)		.06	
	Use of social networking service	30 (41.7)		25 (25.3)		.02	
	Emailing	22 (30.6)		33 (33.3)		.70	
	Internet banking	7 (9.7)		4 (4)		.14	
	Other purposes	4 (5.6)		4 (4)		.64	
**Daily use of digital devices**							
	Cellular phones (other than smartphones)	57 (40.4)		116 (44.1)		.48	
	Smartphones	61 (43.3)		58 (22.1)		<.001	
	Tablet devices	11 (7.8)		13 (4.9)		.25	
	Personal computers	28 (19.9)		40 (15.2)		.23	
**Health information seeking using the internet^a^**							.71
	Yes	24 (17.4)		47 (19)			
	No	114 (82.6)		201 (81)			

^a^Information missing for 3 patients without and 15 patients with cancer.

^b^Patients without cancer n=72, and patients with cancer n=99.

**Table 2:** Proportions of the patients with daily digital device use and regular health information seeking on the internet in patients with cancer and patients without cancer, with classifications of 10-year age unit.

			Cellular phone	Smartphone		Tablet device		Personal computer		Health information collection on the internet
**Patients with cancer (age in years), n (%)**										
	49 or younger (n=28)	4 (14)		23 (82)	2 (7)		13 (46)		13 (46)	
	50-59 (n=45)	20 (44)		17 (38)	4 (9)		17 (38)		16 (36)	
	60-69 (n=79)	42 (53)		13 (16)	5 (6)		5 (6)		12 (15)	
	70-79 (n=73)	39 (53)		3 (4)	2 (3)		5 (7)		5 (8)	
	80-89 (n=38)	11 (29)		2 (5)	0 (0)		0 (0)		1 (3)	
**Patients without cancer, (age in years) n (%)**										
	49 or younger (n=42)	7 (17)		36 (86)	6 (14)		11 (26)		8 (19)	
	50-59 (n=21)	5 (24)		16 (76)	1 (5)		8 (38)		7 (33)	
	60-69 (n=38)	25 (66)		7 (18)	4 (10)		7 (18)		8 (23)	
	70-79 (n=18)	10 (56)		0 (0)	0 (0)		1 (6)		0 (0)	
	80-89 (n=22)	10 (45)		2 (9)	0 (0.0)		1 (5)		1 (5)	

Table 3 shows the findings of the multivariable logistic regression analyses for health information seeking using the internet among patients with cancer and patients without cancer. With regard to family cohabitation, patients with cancer who lived together with at least one family member either pre- or postdisaster tended to seek health information using the internet though a difference was not statistically significant (odds ratio [OR] 0.33, 95% CI 0.09-1.17). However, this was not the case for patients without cancer (OR 0.47, 95% CI 0.10-2.17). With regards to other covariates, patients with cancer who used smartphones (OR 3.73, 95% CI 1.58-8.80) or tablet devices (OR 5.08, 95% CI 1.27-20.35) on a daily basis were significantly more likely to seek health information using the internet. This was also true for patients with cancer who trusted institutional websites (OR 2.87, 95% CI 1.13-7.25). Furthermore, this was also the case for patients with cancer who generally seek information (OR 6.30, 95% CI 2.44-16.29). On the other hand, among patients without cancer, those with no occupation were less likely to seek health information using the internet (OR 0.26, 95% CI 0.08-0.85). In addition, patients without cancer who trusted institutional websites (OR 6.76, 95% CI 2.19-20.88) and personal websites (OR 6.97, 95% CI 1.49-32.58) were more likely to seek health information using the internet.

**Table 3:** Multivariable logistic regression models for health information seeking using the internet among patients with and those without cancera.

Covariates			Patients without cancer (n=136)^b^				Patients with cancer (n=243)^c^		
			OR^d^ (95% CI)		*P* value		OR (95% CI)		*P* value
**Living together with at least one family members either pre- or postdisaster**					.33				.09
	No	Reference				Reference			
	Yes	0.47 (0.10-2.17)				0.33 (0.09-1.17)			
**Employment status**					.03				
	Employed	Reference							
	Not employed	0.26 (0.08-0.85)							
**Daily use of smartphones**									.003
	No					Reference			
	Yes					3.73 (1.58-8.80)			
**Daily use of tablet devices**									.02
	No					Reference			
	Yes					5.08 (1.27-20.35)			
**Reliance on institutional websites**					.001				.03
	No	Reference				Reference			
	Yes	6.76 (2.19-20.88)				2.87 (1.13-7.25)			
**Reliance on personal websites**					.01				
	No	Reference							
	Yes	6.97 (1.49-32.58)							
**General information seeking using the internet**									<.001
	No					Reference			
	Yes					6.30 (2.44-16.29)			

^a^Only the variables which were kept in the final models for the patients with cancer and patients without cancer are presented in the table.

^b^Variation inflation factor of the variables included in the final model ranged from 1.06 to 1.10 and was judged to be sufficiently low.

^c^Variation inflation factor of the variables included in the final model ranged from 1.01 to 1.60 and was judged to be sufficiently low.

^d^OR: odds ratio.

## Discussion

### Principal Findings

Our study assessing both patients with cancer and patients without cancer in the area affected by the Fukushima triple disaster revealed that fewer than 20% of patients with cancer sourced health information using the internet. It should be noted that the proportion was similar among the patients without cancer, with no statistically significant difference detected. Residing with at least one family member before and after the disaster was associated with the internet use for health information seeking among patients with cancer in a clinically meaningful way, though the difference itself was not statistically significant. On the other hand, daily use of smartphones and tablet devices, reliance on newsletters and institutional websites, and general information seeking were positively associated with this behavior. Additionally, younger individuals seeking web-based health information were more reliant on internet-based sources than older individuals.

Studies conducted in the United States between 2007 and 2013 indicated that approximately 60% of patients with cancer search for web-based health information under normal circumstances, a finding that was echoed in a study carried out in Singapore from 2015 to 2016 [5,9,33]. However, in this study, we observed a notably lower percentage of patients with cancer using the internet to gather health information. Several explanations are possible for this discrepancy. First, the median age of the patients with cancer in this study was approximately 67, around 10 years older than in the previous studies. Previous studies have identified older age as a barrier to gathering web-based health information [4,7,9]. It is possible that this is also influencing this study, however, in our multiple variable analysis, this factor did not show a statistically significant effect. This indicates that the age of the participants does not completely explain the lower internet use. Hence, it is likely that our patients are not as active in using the internet for information gathering as patients in previous studies. Second, only a small proportion of our patients with cancer used digital devices suitable for internet use, including smartphones (n=58, 22.1%), tablets (n=13, 4.9%), and personal computers (n=40, 15.2%). Given the significant association between daily digital device use and web-based health information seeking, the limited use of these devices likely contributed to the reduced internet use for health information seeking. Third, our study was conducted in a remote area. Minamisoma City was originally remote before the disaster and its remoteness was exacerbated afterward [25]. Studies have indicated that remoteness predicts less frequent internet use for health information seeking [10], suggesting that the city’s environment may have affected our findings.

We also found that patients with cancer living with family members tended to seek health information on the internet. Although this association was not statistically significant, we considered this difference as clinically meaningful. Our original hypothesis was that the patients’ coliving family members may have helped the patients learn how to use the internet and gain the custom of seeking health information on the internet, and thus, the obtained findings were basically consistent with what we hypothesized. This could be interpreted as patients having extra support in learning how to use the internet from their family members. However, given that there was no significant association between the presence of family members and health information seeking via internet use, the finding does not rule out the possibility that patients with cancer may have gained necessary health information directly from their cohabiting family members simultaneously. Indeed, family members are reported to be key providers of health information for patients with cancer [34], and more likely to seek web-based health-related information, which behavior can even predict family health outcomes [35].

In addition, our results suggested a correlation between patients’ information-gathering habits and their use of digital devices, such as smartphones and tablets, as well as their reliance on institutional websites and newsletters. This connection likely stems from the convenience and accessibility of digital devices. With a smartphone or tablet, information is readily available at any time and place, making them crucial tools for staying informed about one’s health condition. Additionally, the prevalence of these habits indicates a certain level of digital literacy among the patient population, signifying that these patients can navigate web-based resources to gather pertinent information about their condition. The reliance on newsletters and institutional websites also suggests a preference for curated, reputable, and possibly personalized sources of health-related information. The combination of these factors implies that these habits not only allow patients with cancer to stay informed but also provide them with the tools to take an active role in managing their condition.

There were strong age effects on daily digital device use and web-based health information seeking among patients with cancer. In this study, younger patients showed higher engagement in these behaviors, reflecting potentially enhanced access to digital devices and web-based health information. As such, therefore, as reported in another study [36], it is expected that health information seeking via the internet will become more common among patients with cancer in the future, regardless of whether it is during a disaster or a nondisaster situation.

These circumstances urge the relevant stakeholders, including health care professionals, policy makers, and media outlets, to establish proper strategies to deliver necessary health information on the internet including and beyond the aftermath of the 2011 Fukushima triple disaster. Especially, the delivery of information should not be a one-way process but rather requires tailoring and optimization that consider patients’ unique needs, preferences, and digital device use patterns. Moreover, recognizing and implementing these measures not just in crisis situations but also under normal circumstances could serve as a vital approach to enhancing the quality of health care services.

### Limitations

There are several limitations to this study. First, the study assessed data from only a single institution and focused exclusively on patients with breast cancer. This may have introduced a gender bias or cultural background influences, potentially affecting the results. Furthermore, the study was not conducted in the context of comparing conditions before and after the disaster, nor did it evaluate the types of health information that patients with cancer sought on the internet. To gain a comprehensive understanding of the information needs of patients with breast cancer, further research, including qualitative studies, is necessary. Moreover, given that this survey was conducted during 2016-2017, its results might not fully capture the impact of recent advancements in information acquisition such as social media and artificial intelligence chatbots. Although these limitations constrain the generalizability of the findings, it is important to note that the majority of patients with cancer are older people and may not be familiar with these new methods of information gathering [37-39], which is particularly relevant in the context of global aging [40]. Therefore, although the robustness of these insights may have limitations, they are deemed potentially valuable for improving strategies to provide health information in environments affected by disasters, especially in regions with advancing aging populations.

### Conclusions

In this study, we assessed the prevalence and other characteristics of health information seeking among patients with cancer affected by the 2011 Fukushima triple disaster. We primarily found that only limited patients with cancer sought health information on the internet. In addition, factors, such as treatment status, use of digital devices, reliance on institutional websites and newspapers, and living with family, were identified as significant determinants influencing internet use for health information gathering among patients with cancer compared to patients without cancer. Given that both daily digital device use and health information seeking on the internet were more prevalent in the younger population, their prevalence would increase in the future. Considering these patterns and the likelihood of increased internet use for health information in the future, health care professionals, policy makers, and media outlets should think about specific strategies to deliver necessary health information to patients with cancer in postdisaster settings. Therefore, we believe that health care professionals, policy makers, and media outlets should conceive of specific strategies to deliver necessary health information to patients with cancer in postdisaster settings. The present findings should be used to tailor the health information delivery among patients with cancer in disaster settings beyond and including the aftermath of the 2011 Fukushima triple disaster.

## Abbreviations

FDNPP: Fukushima Daiichi nuclear power plant
HIE: health information exposure
MMGH: Minamisoma Municipal General Hospital
OR: odds ratio

## Data Availability

The data sets generated during or analyzed during this study are available from the corresponding author upon reasonable request.
